# A Web-Based Telehealth Training Platform Incorporating Automated Nonverbal Behavior Feedback for Teaching Communication Skills to Medical Students: A Randomized Crossover Study

**DOI:** 10.2196/jmir.6299

**Published:** 2016-09-12

**Authors:** Chunfeng Liu, Renee L Lim, Kathryn L McCabe, Silas Taylor, Rafael A Calvo

**Affiliations:** ^1^School of Electrical and Information EngineeringThe University of SydneySydneyAustralia; ^2^Sydney Medical SchoolThe University of SydneySydneyAustralia; ^3^Psychiatry and Behavioral SciencesUniversity of California, DavisDavis, CAUnited States; ^4^UNSW MedicineUniversity of New South WalesSydneyAustralia

**Keywords:** nonverbal communication, nonverbal behavior, clinical consultation, medical education, communication skills, nonverbal behavior detection, automated feedback, affective computing

## Abstract

**Background:**

In the interests of patient health outcomes, it is important for medical students to develop clinical communication skills. We previously proposed a telehealth communication skills training platform (EQClinic) with automated nonverbal behavior feedback for medical students, and it was able to improve medical students’ awareness of their nonverbal communication.

**Objective:**

This study aimed to evaluate the effectiveness of EQClinic to improve clinical communication skills of medical students.

**Methods:**

We conducted a 2-group randomized crossover trial between February and June 2016. Participants were second-year medical students enrolled in a clinical communication skills course at an Australian university. Students were randomly allocated to complete online EQClinic training during weeks 1–5 (group A) or to complete EQClinic training during weeks 8–11 (group B). EQClinic delivered an automated visual presentation of students’ nonverbal behavior coupled with human feedback from a standardized patient (SP). All students were offered two opportunities to complete face-to-face consultations with SPs. The two face-to-face consultations were conducted in weeks 6–7 and 12–13 for both groups, and were rated by tutors who were blinded to group allocation. Student-Patient Observed Communication Assessment (SOCA) was collected by blinded assessors (n=28) at 2 time points and also by an SP (n=83). Tutor-rated clinical communications skill in face-to-face consultations was the primary outcome and was assessed with the SOCA. We used t tests to examine the students’ performance during face-to-face consultations pre- and postexposure to EQClinic.

**Results:**

We randomly allocated 268 medical students to the 2 groups (group A: n=133; group B: n=135). SOCA communication skills measures (score range 4–16) from the first face-to-face consultation were significantly higher for students in group A who had completed EQClinic training and reviewed the nonverbal behavior feedback, compared with group B, who had completed only the course curriculum components (*P*=.04). Furthermore, at the second face-to-face assessment, the group that completed a teleconsultation between the two face-to-face consultations (group B) showed improved communication skills (*P*=.005), and the one that had teleconsultations before the first face-to-face consultation (group A) did not show improvement.

**Conclusions:**

The EQClinic is a useful tool for medical students’ clinical communication skills training that can be applied to university settings to improve students clinical communication skills development.

## Introduction

There is good evidence that effective patient-clinician communication can positively influence patient health outcomes [1-3]. For instance, a clinician’s supportive expressions can help the patient to develop greater feelings of trust toward their clinician. These feelings of trust lead to greater patient self-efficacy, where the patient is more likely to follow recommended therapies, resulting in a better treatment outcome [4]. This evidence has meant that more training programs are being offered to students to help them learn clinical communication skills. Since students become competent through practice and feedback [5], medical students need practice with real or standardized patients (SPs) and feedback from patients and tutors. An SP normally refers to someone who has been trained to act as a patient in a medical situation. However, despite the importance of communication skills, the time allocated to such training within medical curricula is often limited. This is influenced, in part, by the logistics of providing large groups of medical students with access to SPs with whom they can practice and formulate their communication techniques.

The traditional method for clinical communication skills training is to provide students with feedback on video-recorded face-to-face consultations [6]. Students benefit from reviewing these videotapes of their clinical consultations with real patients or SPs [7], even more so when observers provide feedback about the verbal or nonverbal behaviors [8]. However, organizing large-scale face-to-face practice sessions and setting up the recording environment are a challenge for medical schools. Teleconferencing has been proposed as a solution for dealing with this challenge [9]. For example, the WebEncounter teleconference platform, developed to enable medical interns to communicate with SPs, showed that practicing on WebEncounter enhanced the communication skills of the interns when giving bad news [10]. Another recent study that related to medical students also suggested that involving telehealth consulting between medical practices and patients enhanced students’ learning [11].

However, like WebEncounter, most clinical communication skills training systems tend to limit training to verbal communication skills and overlook important nonverbal communication behaviors. This is problematic, given that nonverbal communication is the major communication channel between individuals [12,13]. Manually annotating students’ nonverbal behaviors from face-to-face consultations and providing this feedback to students are common ways to improve the learning of nonverbal communication skills [14]. However, the practicalities of providing this type of feedback means that it is too time consuming to be widely adopted in medical education curricula.

We have previously described a platform called EQClinic [15,16]. Briefly, EQClinic is an e-learning platform that allows medical students to have recorded teleconsultations with SPs. The platform uses computer vision and audio processing techniques to automatically recognize, quantify, and visualize selected nonverbal behaviors (as well as human feedback) for student learning and reflection. Initial pilot application of EQClinic has shown that medical students’ awareness of their nonverbal communication improved using EQClinic. However, the platform has not been applied within a typical university medical school curriculum.

Therefore, the goal of this study was to conduct a randomized crossover trial of the EQClinic incorporated into a university medical school curriculum (Multimedia Appendix 1 [17]). The EQClinic platform is designed to provide clinical communication skills training that integrates nonverbal behavior assessment for medical students. We used a randomized crossover design to initially test the effectiveness of the EQClinic, whereby we allocated medical students enrolled in a clinical communication skills course to 1 of 2 groups and asked them to complete a teleconsultation using EQClinic at different times during the semester. Interleaved with exposure to the EQClinic were face-to-face clinical consultation skills assessments. By staging exposure of students to the EQClinic, we evaluated the potential impact of the platform by comparing group performance on face-to-face clinical consultations before and after EQClinic exposure. To our knowledge, this is the first application of automated nonverbal behavior detection techniques for improved medical students’ communication skills. We hypothesized that the use of EQClinic would improve medical students’ learning about communication skills.

## Methods

### Participants

Participants were second-year undergraduate medical students from an Australian medical school. All students were enrolled in a communication skills training course provided by the medical school. Prior to this study, they were not offered any training about teleconsultations in the medical school. This study was approved by the University of New South Wales Research Ethics Committee (HC Reference Number: HC16048). Students were asked to sign an online consent form when they first accessed EQClinic. No content or methodological modifications were made after study commencement.

### Questionnaires

The same 5 surveys previously reported by our group were used in the present study [15]. The pre- and postinterview questionnaires ascertained students’ understanding of communication skills. The Post Interview Nonverbal Behavior Reflection Questionnaire asked students to estimate how often they engaged in certain nonverbal behaviors during the interview. The Reflection Questionnaire prompted students to reflect on the consultation. The primary outcomes measure was the Student-Patient Observed Communication Assessment (SOCA) form, which is an adapted version of the Calgary Cambridge Guides [18]. The SPs and tutors used the SOCA to rate students’ communication skills. The form contained four aspects: providing structure, gathering information, building rapport, and understanding the patient’s needs.

### EQClinic

EQClinic comprises five components: an online training component, a personal calendar, a real-time interaction component, a nonverbal behavior detector, and a feedback generator. In the following sections, we briefly describe each of these.

#### Training Component and Personal Calendar

EQClinic provides training videos and documents for students and SPs to familiarize themselves with the platform. EQClinic also provides students and SPs with an automated personal calendar. SPs can offer their availability on the calendar for students to make a booking. All appointments are confirmed using the automated messaging system without need for human resources.

#### Real-Time Interaction Component

Once the appointment has been confirmed, videoconferencing enables a student and an SP to have a teleconsultation. The application works on most Web browsers of a personal computer or Android tablet. During the recorded consultation, the SP can record positive and negative moments using a “thumbs” tool and comment box.

To facilitate learning through reflection, online assessments were included for students. The SPs evaluated student performance immediately after the teleconsultation, during which time the students conduct a self-assessment using the same form. Students could immediately review the SP’s rating.

#### Nonverbal Behavior Detector

Using audio processing and computer vision techniques, EQClinic automatically analyses the video recordings and detects the following nonverbal behaviors: head movements (nodding, head shaking, and head tilting), facial expressions (smiling and frowning), body movements (body leaning, hand gestures, and overall body movements), voice properties (volume and pitch), and speech patterns (turn taking and speaking ratio changes).

#### Feedback Generator

Feedback information includes computer-generated nonverbal behavior feedback (NVBF) and comment feedback from the SP. EQClinic visualizes students’ nonverbal behavior using two types of feedback reports: single-feature and combined-feature reports. The single-feature feedback report illustrates each form of nonverbal behavior separately. The combined-feature feedback report displays multiple kinds of nonverbal behavior on one page. The comment feedback provides students a report that contains all the comments from the SP and tutor.

### Study Design and Procedure

**Figure 1:**
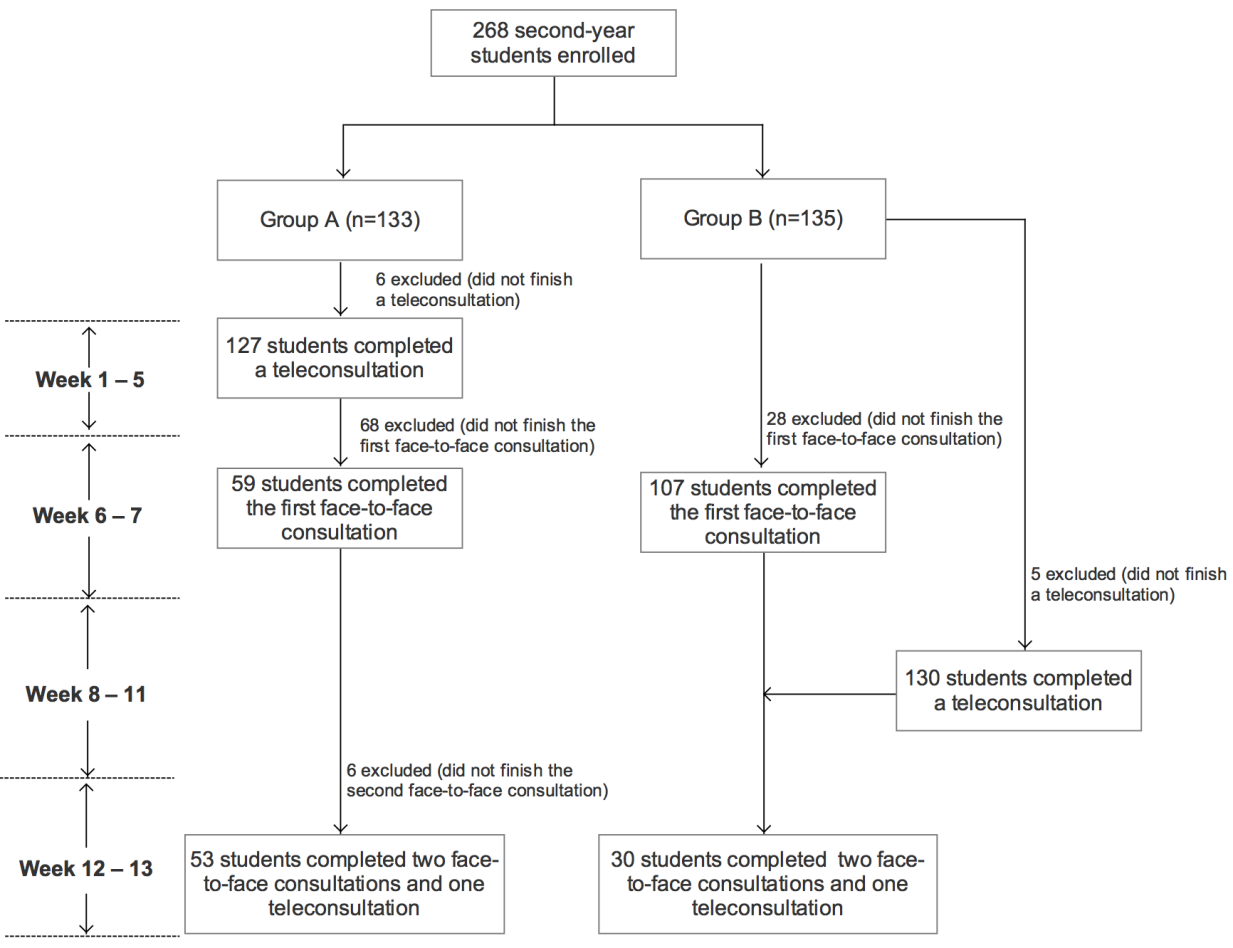
Flowchart of student participation in the EQClinic medical communication training program.

The administrator of this course randomly allocated a cohort of 268 students to group A (n=133) or group B (n=135) (see Figure 1) using a computer-generated random number sequence. One student was moved from group A to group B for administrative reasons. Following random allocation to a group, each participant was provided three opportunities to complete simulated clinical consultations with SPs: a teleconsultation using EQClinic, and two face-to-face consultations. In this study, all consultations focused on history-taking skills, to ensure a structured and consistent interaction. The allocation of the three consultations was varied between the 2 groups. The study was conducted over 13 weeks, and it included 4 periods (see Figure 1). (1) During weeks 1–5, group A completed a teleconsultation using EQClinic and group B was blocked from the platform. (2) During weeks 6 and 7, both groups completed a face-to-face consultation. In this period, group A was still able to access the platform for reviewing feedback only. (3) During weeks 8–11, group B completed an EQClinic consultation and group A was blocked from the platform. (4) During weeks 12 and 13, both groups were asked to complete another face-to-face consultation. In this period, group B was able to access the platform for reviewing feedback only. Due to the limited resources of setting up face-to-face consultations, not all enrolled students completed two face-to-face consultations. However, having a teleconsultation using EQClinic was mandatory for every student.

#### Teleconsultations Procedure

All participating SPs and students completed training via the training component of EQClinic. In the SPs’ online training component, training videos demonstrated how to book appointments, conduct consultations with students, provide comments, and evaluate the student’s performance. The patient scenario was also included in training and detailed the main symptoms of the SP and other historical information. All SPs were required to complete this online training. Following training, the SPs listed their availability for consultations on their EQClinic calendar.

Students were requested, by email, to complete one teleconsultation with an SP through EQClinic. The email described the details of the study and asked them to log in to EQClinic to complete the training module. It also informed them that, once they finished the training, they could request a consultation time from the slots available on their personal calendar. The SPs and students were allowed to have the teleconsultation anywhere as long as there was (1) a Web browser on a personal computer or an Android tablet with an external or built-in camera and microphone, (2) a good Internet connection, and (3) good lighting.

**Figure 2:**
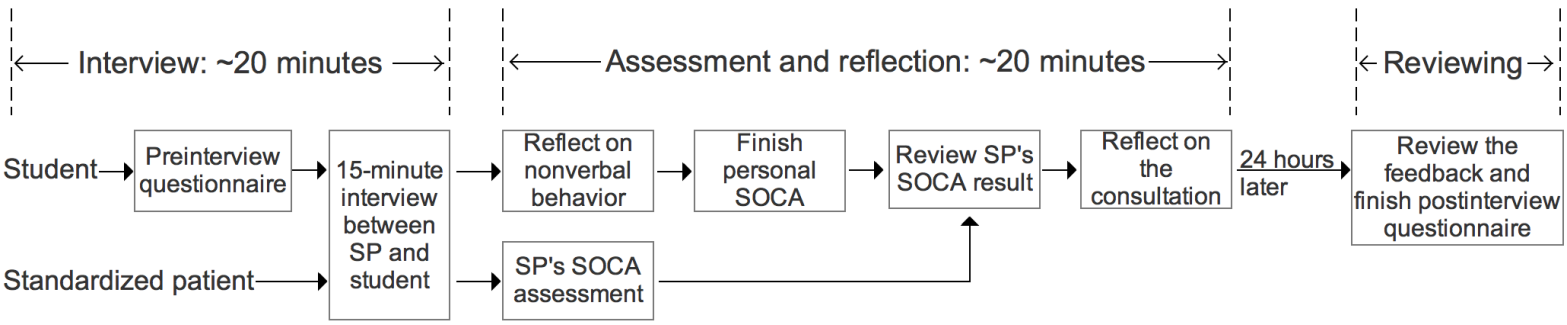
Workflow for the EQClinic consultation. SOCA: Student-Patient Observed Communication Assessment; SP: standardized patient.

EQClinic teleconsultation comprised three sections: interviewing, assessing, and reviewing (see Figure 2) [15]. Interview and assessment components took approximately 40 minutes for a student and 25–30 minutes for an SP to complete. In the interviewing section, the student completed the preinterview questionnaire, and then the student and the SP conducted a 15-minute consultation via the teleconference component. The student and the SP then completed the online assessments. After each interview, the SP assessed the performance of the student using the SOCA form. Meanwhile, the student estimated their nonverbal behavior using the Post Interview Nonverbal Behavior Reflection Questionnaire, completed a personal SOCA form, and then reviewed the SOCA form completed by the SP and reflected on the interview using the Reflection Questionnaire.

Students were emailed to ask them to return to the system 24 hours after the consultation to review different kinds of feedback, which included the video recording, comments from the SP, and automated NVBF. Students also completed the postinterview questionnaire.

#### Face-to-Face Consultations Procedure

Face-to-face consultations were conducted in consultation rooms of a university-based clinical skills center. A trained tutor was present in the room to observe and assess the performance of the student during the consultation with the SP. The tutors were blinded to condition allocation (group A or group B). The tutor completed a SOCA form to assess the student, and the SP did not provide any evaluation and feedback for the student on this occasion. The students were asked to review the tutor’s assessment and complete the Reflection Questionnaire. The scenario design and length of face-to-face consultations were the same as those for the teleconsultations.

## Results

Figure 1 shows participants’ flow through the trial. In the period of week 1 to week 5, 127 (46 male, 81 female) of 133 (95.5%) group A students completed the teleconsultation on EQClinic. In the second period (weeks 6–7), 166 of the 268 students from both group A (59/133, 44.4%) and group B (107/135, 79.3%) completed a face-to-face consultation. During weeks 8–11, a total of 130 (62 male, 68 female) of 135 (96.3%) group B students completed the teleconsultation using EQClinic. Lastly, 144 students from both group A (n=109) and group B (n=35) completed a face-to-face consultation during the period of weeks 12 and 13. In total, 11 students (6 from group A, 5 from group B) did not complete the teleconsultation in this study. At the second face-to-face consultation (weeks 12–13), 53 of 133 (39.9%) students from group A and 30 of 135 (22.2%) from group B had completed one teleconsultation and one face-to-face consultation before completing the second face-to-face consultation.

Table 1 and Table 2 describe the mean subgroup assessment results in the various study periods. Mean total SOCA scores from the first face-to-face consultation for group A (mean 13.02) and group B (mean 12.58) did not differ significantly between the groups (*P*=.08). To examine the influence of the NVBF component, we compared the group A mean SOCA scores (group A + NVBF: mean 13.21; 33/59, 55.9%) of those who had reviewed the NVBF component of the EQClinic before having their face-to-face consultations with the scores of group B students (mean 12.58). Mean SOCA scores were significantly higher on face-to-face SOCA total score in group A + NVBF (t_58.25_=2.13, *P*=.04) than in group B. However, they did not differ statistically from group A students who did not review their nonverbal behavior component of the EQClinic (mean 12.77; 26/59, 44.1%, *P*>.05).

**Table 1:** Mean group medical communication skills (measured by Student-Patient Observed Communication Assessment score) assessment results (part 1: weeks 1–7).

Component	Weeks 1–5 (TC^a^)		Weeks 6–7 (F2FC^b^)					
	Group A (n=127)		Group A (n=59)		Group A (NVBF^c^) (n=33)		Group B (n=107)	
	Mean	SD	Mean	SD	Mean	SD	Mean	SD
Total score	11.59	2.67	13.02	1.49	13.21	1.45	12.58	1.61
Providing structure	2.88	0.72	3.17	0.50	3.27	0.45	3.12	0.53
Gathering information	2.92	0.72	3.25	0.58	3.15	0.51	3.07	0.56
Building rapport	2.95	0.73	3.34	0.60	3.39	0.66	3.24	0.56
Understanding patient’s needs	2.83	0.80	3.25	0.58	3.39	0.56	3.14	0.61

^a^TC: teleconsultation.

^b^F2FC: face-to-face consultation.

^c^NVBF: students who had a face-to-face consultation and reviewed the nonverbal behavior feedback.

**Table 2:** Mean group medical communication skills (measured by Student-Patient Observed Communication Assessment score) assessment results (part 2: weeks 8–13).

Component	Weeks 8–11 (TC^a^)		Weeks 12–13 (F2FC^b^)									
	Group B (n=130)		Group A (n=109)		Group A (ConA^c^) (n=53)		Group B (n=35)		Group B (ConB^d^) (n=30)		Group B (NVBF^e^) (n=13)	
	Mean	SD	Mean	SD	Mean	SD	Mean	SD	Mean	SD	Mean	SD
Total score	13.13	2.31	13.28	1.54	13.28	1.46	13.43	1.63	13.53	1.52	13.62	1.64
Providing structure	3.31	0.67	3.25	0.49	3.23	0.46	3.31	0.52	3.37	0.48	3.38	0.49
Gathering information	3.34	0.72	3.26	0.55	3.30	0.53	3.51	0.55	3.53	0.50	3.46	0.50
Building rapport	3.16	0.73	3.46	0.58	3.47	0.54	3.34	0.58	3.37	0.55	3.46	0.63
Understanding patient’s needs	3.32	0.68	3.31	0.57	3.28	0.59	3.26	0.44	3.27	0.44	3.31	0.46

^a^TC: teleconsultation.

^b^F2FC: face-to-face consultation.

^c^ConA: group A students who participated in two consultations (one face-to-face consultation, one teleconsultation) before week 12.

^d^ConB: group B students who participated in two consultations (one face-to-face consultation, one teleconsultation) before week 12.

^e^NVBF: students who had a face-to-face consultation and reviewed the nonverbal behavior feedback.

Following group B exposure to the EQClinic, the mean total SOCA scores from the second face-to-face consultation did not differ between the groups (group A: mean 13.28; group B: mean 13.53, *P*>.05). Mean SOCA scores of group B students (group B + NVBF: mean 13.62; 13/30, 43.3%) who reviewed the NVBF component of the EQClinic before their second face-to-face consultation did not differ from those in group A or group B who did not complete the nonverbal review (mean 13.47; 17/30, *P*>.05).

We used paired-samples *t* tests to compare the SOCA assessment scores for those students who completed EQClinic on both their two face-to-face consultations. Group B alone showed significant improvement in their mean SOCA score (mean preexposure score 12.58 vs postexposure score 13.53; t_48_= –2.96; *P*=.005). Group A showed no significant increase in SOCA scores (mean preexposure score 13.02 vs postexposure score 13.28; *P*>.05). Comparison of the mean SOCA teleconsultation scores rated by SPs showed that group B’s score (mean 13.13) was significantly higher than group A’s score (mean 11.59; t_246.61_= –4.83, *P*<.001).

## Discussion

We incorporated EQClinic into a medical communication skills teaching curriculum to provide students with additional practice opportunities with SPs. Importantly, students could review their nonverbal communication behaviors. We examined the effects of EQClinic on medical students’ learning of communication skills evaluated via the students’ assessment (SOCA) scores. Results showed that students who completed a teleconsultation using EQClinic and reviewed the NVBF achieved higher SOCA scores in the first face-to-face consultation. In addition, students accomplished higher SOCA scores in their second face-to-face consultation if they completed a teleconsultation between the two face-to-face consultations.

Overall, adherence to the program was somewhat less than anticipated, with only 30% of student completing all components of the study. Dropout increased as the semester progressed. However, given the requirements of the undergraduate course, and the tendency for increased workload as the semester progresses, this result is unsurprising.

The results of the first face-to-face consultation show that the students who completed a teleconsultation and reviewed the NVBF component scored significantly higher in their face-to-face consultation than did students who did not interact with SPs on EQClinic. These results are promising. The difference in performance between the 2 groups seems to indicate that having EQClinic practice coupled with reviewing feedback improved medical communication skills in group A. As noted above, group B students achieved lower mean overall SOCA score in the first face-to-face consultation. However, overall, group B students showed significant improvement from their first to second face-to-face consultation. However, whether students reviewed their NVBF did not influence results for this group.

These findings are interesting because they suggest improvement in communication skills assessment after reviewing nonverbal feedback. While the need for medical communication skills training is widely accepted within the medical teaching community [1-3], there is less consensus on the need for specific teaching on the nonverbal aspects of communication. This is related to the lack of adequate resources, knowledge, and expertise in this aspect of communication [19]. To our knowledge, this is the first study to systematically incorporate nonverbal learning feedback into medical communication skills training.

Furthermore, that we showed no significant difference between group scores in the second face-to-face assessment seems to indicate that the timing of exposure to EQClinic within a teaching curriculum did not influence students’ learning results. In our study, group A was exposed to EQClinic at the beginning of the course; whereas group B was exposed in the middle of their course. We showed that at the commencement of the curriculum, when students did not have significant knowledge of clinical communication skills, exposure to EQClinic yielded a measurable bump in their clinical communication skills. For medical educators this seems to indicate that EQClinic could be incorporated at any period during the teaching curriculum.

We also showed that group B performed significantly better than group A on the SP-rated EQClinic teleconsultations. This difference could be explained in several ways. The first way relates to timing of EQClinic exposure, with group B completing the teleconsultation later in the semester than group A. Second, completing face-to-face consultations before being exposed to EQClinic, experience, and feedback garnered from the face-to-face consultation may have improved student performance in the teleconsultation. The third possibility is that the SPs who assessed students via the EQClinic increased their ratings across the semester. However, SP ratings neither contributed to student assessment nor were a central feature of the EQClinic.

Telehealth studies involving medical students and interns in urban, rural, and remote areas indicated that this medium was a useful learning tool [10,11]. In EQClinic, we enhance existing telehealth systems by providing students with multiple kinds of feedback. We contend that the primary functions of EQClinic are 2-fold: to facilitate student access to SPs to practice and refine their medical communication skills. The importance of SPs to facilitate the application of clinical communication theory, especially early one-on-one interactions, has been described previously [20]. The second function of the EQClinic is to facilitate reflective practice by providing human and computer-generated feedback, in particular in regard to nonverbal behaviors, in medical communication skills training.

However, based on our findings, it remains unclear which of the learning components were most useful to enhancing students’ learning. Moreover, although a single exposure to the EQClinic led to a measurable improvement in students’ medical communication skills scores, future studies will benefit from an examination of the appropriate “dose” of EQClinic. This will help determine the necessary exposure needed to provide sustained improvement and generalizable communication skills training. Finally, the growth of collected student data by EQClinic will aid the refinement of rules and models using machine learning algorithms to indicate to students what nonverbal behavior is associated with positive or negative responses and feedback from SPs in their clinical teleconsultations.

### Study Limitations

There are several limitations to our study that should be considered when interpreting these findings. First, the absence of baseline measures limited our ability to observe change over time. Second, all the consultations conducted in this study were limited to a history-taking scenario. In reality, clinicians encounter many different scenarios. For example, when breaking bad news to patients, the clinician has to handle difficulties related to emotions. In addition, all the students in this study were second-year medical students who had limited knowledge about communication skills. Future studies may explore whether EQClinic is also useful for senior medical students and professionals. A third limitation is the relatively low proportion of students (30%) who completed all components of the study. While the sample was still appropriate for the statistical tests conducted, future investigations will benefit from exploring in greater detail the reason for student nonparticipation.

### Conclusions

This study provided evidence that furnishing medical students with opportunities to conduct teleconsultations with SPs improved medical communication skills. In particular, offering enhanced and quantified feedback information facilitates their reflection and enhances their learning of clinical communication skills. Importantly, this study demonstrated that EQClinic was a useful and practical communication skills learning tool that is well suited to medical students within university settings.

## Multimedia Appendix 1

CONSORT-EHEALTH Checklist V1.6 [20].

## Abbreviations

NVBF: nonverbal behavior feedback
SOCA: Student-Patient Observed Communication Assessment
SP: standardized patient
